# Quantum imaging with ultra-thin metasurfaces

**DOI:** 10.1038/s41377-025-01830-0

**Published:** 2025-04-02

**Authors:** Jongwon Lee

**Affiliations:** Department of Electrical Engineering, Ulsan National Institute of Science and Technology (UNIST), Ulsan, 44919 Republic of Korea

**Keywords:** Metamaterials, Imaging and sensing, Quantum optics

## Abstract

Nonlinear optical metasurfaces, which relax the phase-matching constraints of bulk nonlinear crystals and allow for precise engineering, are opening new possibilities in the field of quantum photonics. Recent advancements have experimentally demonstrated high-resolution 2D imaging using a 1D detector array by combining quantum ghost imaging and all-optical scanning with spatially entangled photon pairs generated from a nonlinear metasurface. These findings establish metasurfaces as a promising platform for quantum imaging, communications, and sensing applications.

Quantum imaging systems utilizing spatially entangled photon pairs demonstrate the potential to achieve high resolution and high sensitivity, surpassing traditional classical optical methods^1–3^. Various applications, such as quantum ghost imaging^4^, imaging with undetected photons^5^, and quantum super-resolution imaging^6^, have been reported. Spatially entangled photon pairs exhibit strong correlations in their spatial coordinates and momentum and are typically generated through the Spontaneous Parametric Down-Conversion (SPDC) process in nonlinear materials, where a high-energy pump photon splits into two lower-energy entangled photons (signal and idler). However, in conventional bulk nonlinear optical crystals, SPDC photon emission is constrained by the longitudinal phase matching condition, which significantly limits the field of view (FOV) for imaging applications^7^.

Nonlinear optical metasurfaces consist of two-dimensional arrays of subwavelength-scale engineered structures that exhibit nonlinear optical responses. They offer advantages over bulk nonlinear crystals, such as relaxed phase-matching constraints and the ability to control nonlinear responses at the subwavelength scale, enabling the manipulation of the wavefront and polarization state of the output beam^8,9^. Recently, studies on quantum light sources with engineered multifrequency quantum states utilizing nonlinear metasurfaces with the SPDC process have also been reported^10–16^. Metasurface-based quantum light sources that generate the SPDC process are primarily investigated in the visible and near-infrared regions, where single-photon detectors necessary for precise coincidence counting are available. Engineered structures based on materials with a large second-order nonlinear response, such as III-V semiconductors^10,12^ or lithium niobate^11,13–15^, have been reported. To achieve an enhanced SPDC process, localized Mie-type optical resonances and nonlocal guided-mode resonances in nanostructured metasurfaces can be utilized to induce strong light-matter interactions. Compared to localized Mie-type resonances, which are determined by the size, shape, and material composition of individual nanostructures, nonlocal guided-mode resonances arise from the coupling of incident light to guided modes that extend over large areas. As a result, they exhibit a relatively high quality-factor and are strongly influenced by incident angle, wavelength, and structural periodicity^13^.

In a recently published paper in *e-Light*, a team led by Prof. Andrey A. Sukhorukov presented research findings on quantum imaging of two-dimensional objects using spatially entangled photon pairs generated from a nonlocal nonlinear metasurface composed of a one-dimensional grating structure^17^. This study represents the first demonstration of utilizing nonlinear metasurface-based quantum photonic sources for quantum imaging. In this research, the team employed a lithium niobate metasurface with a subwavelength-scale silica metagrating to achieve enhanced SPDC for the generation of spatially entangled photon pairs. The unique properties of this metasurface enabled a hybrid quantum imaging approach: (1) Quantum ghost imaging along the z-direction (parallel to the grating stripes, cf. Fig. 1a) was realized using the broad anti-correlated emission pattern of entangled photon pairs. (2) All-optical scanning along the y-direction (perpendicular to the grating stripes, cf. Fig. 1a) was achieved by tuning the photon emission angles through adjustments to the pump laser wavelength, based on the dispersion-dependent behavior of the nonlocal metasurface. Based on these characteristics, the experimental implementation demonstrated the feasibility of high-resolution 2D imaging using a 1D detector array, utilizing the optical setup shown in Fig. 1a. The signal photons generated from the metasurface pass through a 2D object and are detected by a bucket Single Photon Detector (SPD), while the idler photons are detected by an SPD array along the z-direction. By measuring coincidences as a function of the photon wavelengths, 2D imaging data can be reconstructed. Figure 1b presents the results of 2D quantum imaging, combining ghost imaging for z-pixels and optical scanning imaging for y-pixels.

**Fig. 1:**
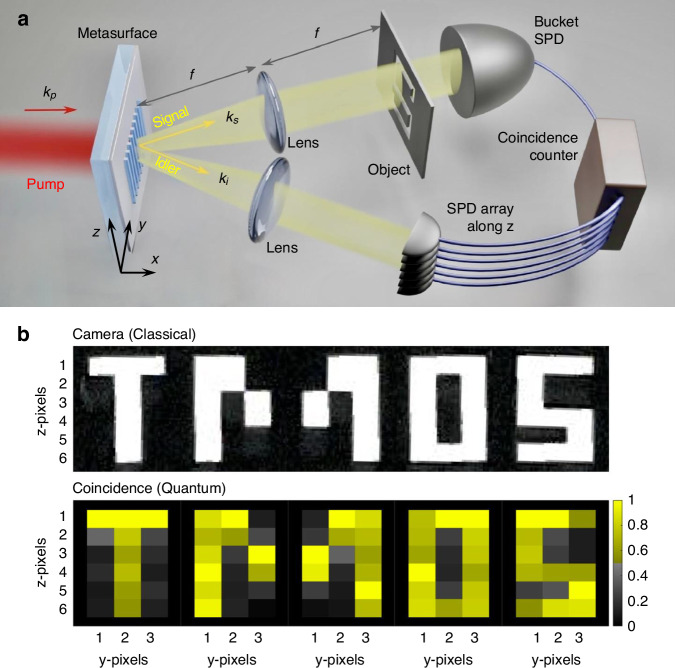
**a** Optical setup for quantum imaging with spatially entangled photon pairs from a nonlinear metasurface. 2D imaging of the object was obtained via quantum ghost imaging and all-optical scanning in the z- and y-direction, respectively. **b** Experimental data of 2D quantum imaging combining ghost imaging and optical scanning. The top and bottom panel show the optical camera image of objects and the reconstructed image from coincidence measurement, respectively^17^

When using traditional nonlinear crystals, the angular range of photon emission is constrained by longitudinal phase-matching conditions. However, by utilizing metasurfaces, these limitations are overcome, enabling a much broader photon emission range and, consequently, a significantly larger FOV, which is one of the key advantages of this approach. Additionally, metasurfaces provide higher imaging resolution by allowing spatially engineered photon entanglement, leading to an image resolution improvement of over four orders of magnitude compared to conventional bulk nonlinear crystals. Another critical advantage is the compact and integrated design of the subwavelength-thick metasurface, which enables seamless integration into photonic circuits and facilitates the development of miniaturized quantum imaging devices.

This advancement highlights the significant role of metasurfaces not only for achieving a practical level of quantum imaging but also for quantum communications and sensing applications. The next step toward practical implementation is improving photon-pair generation rates, which can be achieved through the use of highly nonlinear materials (e.g., III-V semiconductors, ferroelectric materials, and 2D materials) and metasurfaces designed with triple optical resonances for the pump, signal, and idler photons. While this study utilized degenerate signal and idler photon pairs with the same wavelength, it is expected that the approach can be extended to applications such as quantum imaging and sensing with undetected photons by employing nanoantenna structures with multiple resonances to generate non-degenerate photon pairs at different wavelengths. Furthermore, if metasurfaces enabling optical beam steering via pump wavelength tuning or electrical beam steering are utilized, they could open new possibilities for ultrafast quantum LiDAR and real-time object tracking technologies^18,19^.
